# The antihypertension drug doxazosin inhibits tumor growth and angiogenesis by decreasing VEGFR-2/Akt/mTOR signaling and VEGF and HIF-1α expression

**DOI:** 10.18632/oncotarget.2064

**Published:** 2014-06-06

**Authors:** Mi Sun Park, Boh-Ram Kim, Seung Myung Dong, Seung-Hoon Lee, Dae-Yong Kim, Seung Bae Rho

**Affiliations:** ^1^ Research Institute, National Cancer Center, 323, Ilsan-ro, Ilsandong-gu, Goyang-si Gyevonggi-do, Republic of Korea; ^2^ Department of Veterinary Pathology, College of Veterinary Medicine, Seoul National University, 599, Gwanank-ro, Gwanakgu, Seoul, Republic of Korea; ^3^ Department of Life Science, Yong In University, 470, Samga-dong, Cheoin-gu, Yongin-si Gyeonggi-do, Republic of Korea

**Keywords:** doxazosin, anti-angiogenic activity, VEGFR-2, Akt/mTOR phosphorylation, endothelial cell

## Abstract

Doxazosin is an α1 adrenergic receptor blocker that also exerts antitumor effects. However, the underlying mechanisms by which it modulates PI3K/Akt intracellular signaling are poorly understood. In this study, we reveal that doxazosin functions as a novel antiangiogenic agent by inhibiting vascular endothelial growth factor (VEGF)-induced cell migration and proliferation. It also inhibited VEGF-induced capillary-like structure tube formation *in vitro*. Doxazosin inhibited the phosphorylation of VEGF receptor-2 (VEGFR-2) and downstream signaling, including PI3K, Akt, 3-phosphoinositide-dependent protein kinase 1 (PDK1), mammalian target of rapamycin (mTOR), and hypoxia-inducible factor 1 (HIF-1α). However, it had no effect on VEGF-induced extracellular signal-regulated kinase 1/2 (ERK1/2) phosphorylation. Furthermore, doxazosin reduced tumor growth and suppressed tumor vascularization in a xenograft human ovarian cancer model. These results provide evidence that doxazosin functions in the endothelial cell system to modulate angiogenesis by inhibiting Akt and mTOR phosphorylation and interacting with VEGFR-2.

## INTRODUCTION

Angiogenesis is an important but complex process that occurs during endothelial cell development, growth, and movement, as well as wound healing and endothelial cell-mediated degradation of the extracellular matrix. Multistep angiogenesis is vital for cell division and metastasis in most solid tumors. Angiogenesis, the constitution of new blood vessels forming from pre-existing vessels, occurs during embryonic development as well as invasive tumor growth and tumor pathogenesis [1, 2]. Angiogenesis is activated by various signaling molecules and growth factors, including fibroblast growth factor (FGF), transforming growth factor beta (TGF-β), and vascular endothelial growth factor (VEGF). VEGF is a major modulator of endothelial cell function, such as blood vessel formation during embryonic development, and plays a vital role in the proliferation, migration, and invasion of vascular endothelial cells [3]. Angiogenesis is initiated by growth factors such as VEGF and is a potential treatment for vascular injuries [4-6]. During cancer, endothelial cell activity plays an essential role in modulating various vascular physiological and pathological functions. Although VEGF receptor 1 (VEGFR-1) and VEGFR-2 are structurally very similar, they have different biological roles. For example, VEGFR-1 negatively regulates embryonic vasculogenesis and stimulates tumor angiogenesis by activating macrophages, whereas VEGFR-2 is predominantly responsible for both tumor angiogenesis and embryonic vasculogenesis [7-10]. VEGFR-2 also plays a pivotal role in the activation of downstream components that are responsible for proliferation, including endothelial cell invasion, migration, differentiation, and embryonic angiogenesis [11-13]; in contrast, VEGFR-1 has no role in endothelial cell proliferation [14].

Doxazosin (1-[4-amino-6,7-dimethoxy-2-quinazolinyl]-4-[1,4-benzodioxan-2-ylcarbonyl] piperazine methanesulfonate), a quinazoline compound, functions as an α1 adrenergic receptor blocker [15]. It is used to treat patients with benign prostatic hyperplasia, a noncancerous enlargement of the prostate gland, because α1-blockers relax the smooth muscles surrounding the prostate, easing urine flow and decreasing bladder outlet obstruction [16, 17]. Some previous studies also reported that α1-adrenoceptor antagonists stimulate apoptotic cell death via TGF-β1 signaling and the activation of clear factor of kappa light polypeptide gene enhancer in B-cells inhibitor-alpha (IκBα) in prostate tumor cells [16, 18]. Doxazosin also stimulates apoptotic cell death in response to abnormal cell-matrix interactions [19]. In a recent study, Garrison and Kyprianou reported that specific caspase-8 inhibitors could block doxazosin-induced apoptotic cell death in benign prostate cells (BPH) [20]. Caspase-8 is activated by its interaction with Fas-associated death domain (FADD) and their subsequent recruitment by Fas ligand. Nevertheless, the biological roles of these doxazosin-regulated processes, as well as molecular mechanism behind the antiangiogenic effects of doxazosin, remain poorly understood. Endothelial cells play a vital role in regulating various vascular biological effects and related diseases, including tumor growth and maintenance. However, the effects of doxazosin in endothelial cells during ovarian tumor growth are unknown.

Most studies on angiogenesis have focused on endothelial cell proliferation, migration, and capillary-like tubule formation. In the current study, we investigated the antiangiogenic effects of doxazosin in tumors using *in vitro* human umbilical vein endothelial cell (HUVEC) and *in vivo* mouse model systems. We also identified the molecular pathways responsible for Akt- and mammalian target of rapamycin (mTOR)-dependent endothelial cell growth during tumorigenesis. Doxazosin inhibited the phosphorylation of signaling modulators downstream of phosphatidylinositol-3'-kinase (PI3K) including Akt, phosphoinositide-dependent protein kinase 1 (PDK1), and mTOR by interacting directly with VEGFR-2. Therefore, this interaction results in potent antiangiogenic and antitumor effects. The current findings suggest that doxazosin plays a vital role in regulating cellular angiogenesis.

## RESULTS

### Doxazosin suppresses VEGF-induced cell migration, proliferation, and capillary-like tubule formation significantly in HUVECs

**Figure 1:**
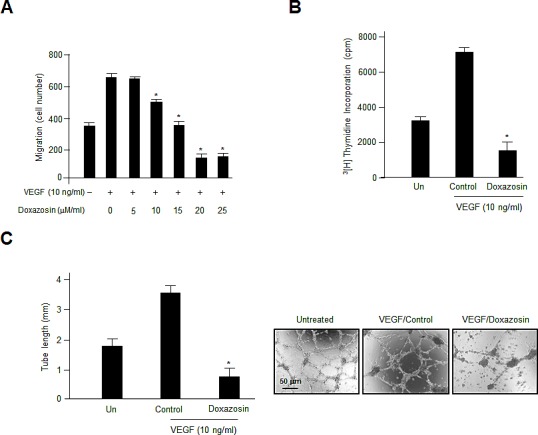
Treatment with doxazosin decreases endothelial cell migration, proliferation, and capillary-like tubule formation (A) The effect of doxazosin treatment on VEGF-stimulated endothelial cell migration was assessed using Boyden Transwell chambers. Cells were treated with increasing concentrations of doxazosin, fixed, and then stained with H&E. The numbers of migrated cells were calculated under a light microscope. Three independent experiments were assayed in triplicate. Data are presented as the means ± SDs. *P<0.05 vs. the control group. (B) The inhibitory effects of doxazosin on endothelial cell proliferation. Cells were incubated for 3 days with or without VEGF. The c.p.m. of [^3^H] thymidine was evaluated using a liquid scintillation counter. The data are presented as the means ± SDs of three independent experiments. *P<0.05 vs. the control group. (C) Cells were treated with doxazosin and then grown on growth factor-reduced Matrigel in the presence or absence of 10 ng/ml of VEGF. Tubular-like structure formation was monitored using inverted microscopy. Tube length was quantified and expressed as means ± SD. Experiments were repeated three times, and representative data are shown. *P<0.05 vs. control.

The migration of endothelial cells is essential during angiogenesis. Therefore, we explored whether doxazosin modulated the effects of VEGF on cell migration using a modified Boyden Transwell chamber system in HUVECs. As expected, VEGF promoted the migration of doxazosin-treated cells compared to control cells. Doxazosin inhibited migration in a dose-dependent manner, and the maximal effect was seen at a concentration of 20 μM (Fig. 1A). No migration was observed after 24 h of treatment with increasing concentrations (0-25 μM) of doxazosin (data not shown). These results suggest that VEGF-stimulated endothelial cell migration and angiogenesis might be inhibited specifically by doxazosin.

The *in vitro* antiangiogenic activity of doxazosin was analyzed by assessing its effects on the VEGF-stimulated proliferation of endothelial cells using [^3^H] thymidine incorporation. Doxazosin inhibited VEGF-induced HUVEC DNA synthesis significantly (Fig. 1B). This antiproliferative effect was not due to the cytotoxicity of doxazosin in endothelial cells, since doxazosin had no effect on the viability of HUVECs, as assessed using Trypan Blue exclusion (data not shown). These results suggest that doxazosin regulates angiostasis and potently inhibits VEGF-induced pivotal events during angiogenesis, including endothelial cell proliferation and migration *in vitro*.

We next assessed the antiangiogenic effects of doxazosin on VEGF-induced capillary-like tubule formation using HUVECs grown on Matrigel *in vitro*. As shown in Fig. 1C, treatment with VEGF increased the formation of extended, strong capillary-like tubular structures compared with control cells. In contrast, doxazosin treatment abrogated VEGF-induced capillary-like tubule formation. Collectively, these results strongly suggest that doxazosin specifically regulates VEGF-induced angiogenesis *in vitro*.

### Doxazosin suppresses PI3K and Akt phosphorylation in a concentration-dependent manner

**Figure 2:**
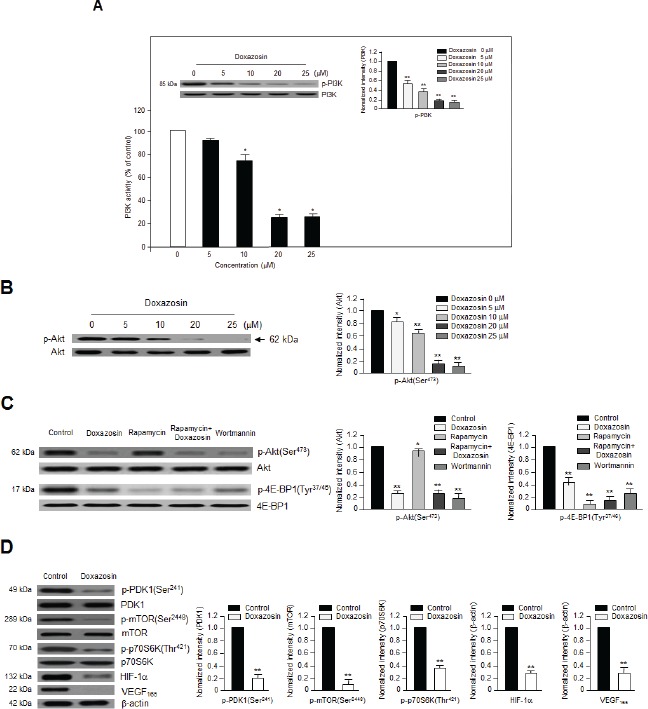
Doxazosin downregulates PI3K activity and Akt/mTOR/p70S6K phosphorylation *in vitro* (A) SKOV-3 cells were treated with increasing concentrations of doxazosin, harvested, lysed, and analyzed using an *in vitro* PI3K assay (lower panel) and immunoblotting with anti-p85 polyclonal antibodies; PI3K was used to verify equal sample loading (upper panel). Data are representative of three independent experiments. Protein levels were calculated by densitometric analysis and normalized to levels of the loading control. *, *P*<0.05; **, *P*<0.01 compared with the controls. (B) SKOV-3 cells were treated with various concentrations of doxazosin, and Akt phosphorylation was evaluated using Western blotting. Protein levels were calculated by densitometric analysis and normalized to levels of the loading control. The data are presented as the means ± SDs of three independent experiments. *P<0.05; **, *P*<0.01 vs. the control group. (C) SKOV-3 cells were treated with doxazosin, the mTOR inhibitor rapamycin, rapamycin plus doxazosin, or the PI3K inhibitor wortmannin. After 24 h, the cells were collected and then analyzed using immunoblotting; total Akt and 4E-BP1 were used as loading controls. The data are presented as the means ± SDs of three independent experiments. Protein levels were calculated by densitometric analysis and normalized to levels of the loading control. *P<0.05; **, *P*<0.01 vs. the control group. (D) After treatment with doxazosin, cells were harvested and analyzed by Western blotting with antibodies against phosphorylated and total PDK1, mTOR, and VEGF and its downstream targets including p70S6K and HIF-1α. β-Actin was used as a loading control. Data are representative of three independent experiments. Protein levels were calculated by densitometric analysis and normalized to levels of the loading control. **, *P*<0.01 compared with the controls.

PI3K/Akt signaling plays a key role during tumor angiogenesis. Therefore, we evaluated whether doxazosin decreased the phosphorylation of PI3K and Akt in SKOV-3 and OVCAR-3 (data not shown) ovarian carcinoma cells. Lysates from SKOV-3 cells treated with various concentrations (0-25 μM) of doxazosin were analyzed by Western blotting, which revealed that VEGF induced the phosphorylation of PI3K and Akt. As shown in Fig. 2A, the activity of PI3K treated with various concentrations of doxazosin inhibited in a dose-dependent manner, the maximum effect was observed with 20 μM. Consistent with this result, 20 μM doxazosin also decreased PI3K phosphorylation significantly. Akt is an essential downstream component of PI3K signaling. As expected, doxazosin gradually reduced Akt phosphorylation in a dose-dependent manner (Fig. 2B). Similarly, doxazosin also lowered Akt phosphorylation in HUVECs (data not shown). Collectively, these results suggest that PI3K/Akt-dependent signaling cascades play important roles in the effects of doxazosin in endothelial cells. The magnitude of the observed effects was comparable to those exerted by well-known PI3K and mTOR inhibitors such as wortmannin and rapamycin, respectively.

In addition to Akt, doxazosin also decreased the phosphorylation of eukaryotic translation initiation factor 4E binding protein 1 (4E-BP1) (Tyr37/46) (Fig. 2C) and PDK1 (Ser241) (Fig. 2D), one of the best-characterized targets of mTOR. When cells were co-treated with doxazosin and rapamycin, p-4E-BP1 was inactivated. These results provide evidence to support our hypothesis that doxazosin inhibits PI3K/Akt activity.

**Figure 3:**
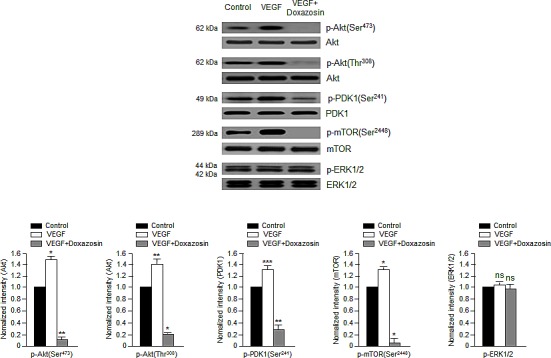
Doxazosin suppresses VEGF-dependent Akt, PDK1, and mTOR phosphorylation SKOV-3 cells were treated with doxazosin and then harvested for immunoblotting with antibodies against phosphorylated or total Akt, PDK1, mTOR, and ERK1/2. The data are presented as the means ± SDs of three independent experiments. Protein levels were calculated by densitometric analysis and normalized to levels of the loading control. *P<0.05; **, *P*<0.01; ***, *P*<0.005; ns, no significant vs. the control group.

We next assessed the effects of doxazosin on downstream mediators of the PI3K/Akt pathways. Doxazosin dramatically diminished the phosphorylation of mTOR at Ser-2448 and p70 ribosomal S6 kinase (p70S6K) at Thr-421 (Fig. 2D). Therefore, we next determined whether doxazosin led to VEGF-induced activation of *the* Akt/PDK1/mTOR complex. As shown **in** Fig. 3, doxazosin decreased the VEGF-induced phosphorylation of Akt (Ser-473 and Thr-308), PDK1 (Ser-241), and mTOR (Ser-2448), but had no effect on the phosphorylation of extracellular signal-regulated kinase 1/2 (ERK1/2). These results strongly suggest that doxazosin decreases VEGF-dependent Akt, PDK1, and mTOR phosphorylation.

**Figure 4:**
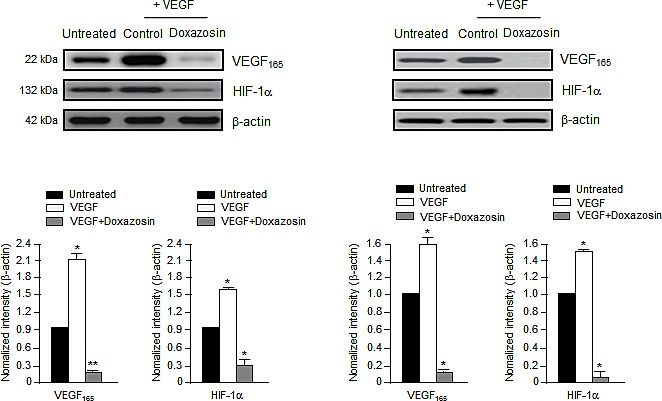
Doxazosin inhibits VEGF-induced HIF-1α and VEGF Cells were incubated with 10 ng/ml VEGF and then control-treated or treated with doxazosin in HUVECs (left panel) or SKOV-3 ovarian carcinoma cells (right panel). HIF-1α and VEGF protein expression levels were then analyzed using Western blotting. Data are representative of three independent experiments. Protein levels were calculated by densitometric analysis and normalized to levels of the loading control. *, *P*<0.05; **, *P*<0.01 compared with the controls.

PI3K/Akt signaling not only mediates VEGF-induced cell proliferation and migration, but also the expression of VEGF and HIF-1α [17]. Consistent with these findings, doxazosin treatment abrogated VEGF and HIF-1α expression completely. These results suggest that VEGF-induced protein activation is inhibited specifically by doxazosin in HUVECs (left panel) and ovarian carcinoma cells (right panel) (Fig. 4). Collectively, these observations suggest that doxazosin inhibits the autocrine effects of VEGF in endothelial cells, exerts direct antiangiogenic effects, and inhibits tumor growth and metastasis.

### Doxazosin inhibits VEGF-induced VEGFR-2 phosphorylation and VEGFR-2-dependent transcription

**Figure 5:**
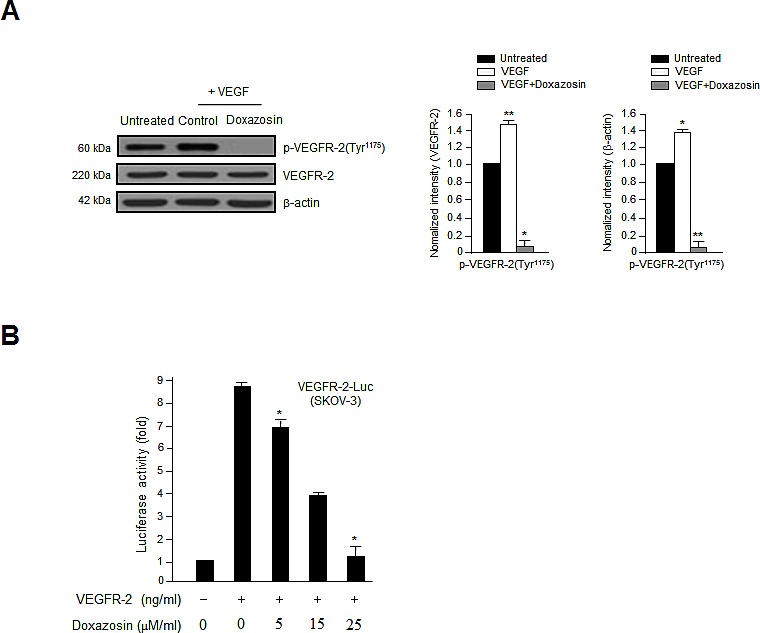
Doxazosin inhibits VEGF-induced VEGFR-2 phosphorylation and VEGFR-2-dependent transcription (A) SKOV-3 ovarian carcinoma cells were incubated with 10 ng/ml VEGF followed by control or doxazosin. Total cell lysates were prepared, and VEGFR-2 phosphorylation was assessed using immunoblotting. Total VEGFR-2 and β-actin were used as loading controls. Data are representative of three independent experiments. *, *P*<0.05; **, *P*<0.01 compared with the controls. (B) The suppression of VEGFR-2-dependent transcription by doxazosin. Cells were co-transfected with 500 ng of VEGFR-2-Luc, 500 ng of VEGFR-2 expression plasmid (pcDNA3.1/VEGFR-2), and increasing concentrations of doxazosin (0, 5, 15, and 25 μM/ml). Data are presented as means ± SDs.*P<0.05 vs. the control.

We next assessed the effect of doxazosin on VEGF-induced VEGFR-2 phosphorylation in SKOV-3 cells and HUVECs (data not shown) to assess the biological and functional relevance of the direct relationship between VEGFR-2 and doxazosin. VEGFR-2 is a key signal transducer during VEGF-induced endothelial cell vascular development and pathological angiogenesis. As shown in Fig. 5A, treatment with doxazosin suppressed VEGF-induced VEGFR-2 phosphorylation in SKOV-3 carcinoma cells. The effect of doxazosin on *VEGFR-2* transcription was then evaluated using a luciferase reporter assay system and a construct containing the *VEGFR-2* promoter fused to *luciferase*. Luciferase activity decreased in response to treatment with doxazosin in a dose-dependent manner (Fig. 5B), suggesting that doxazosin regulates VEGFR-2 activity. These results suggest that treatment with doxazosin decreases *VEGFR-2* transcriptional activity.

### Doxazosin inhibits tumor growth via antiangiogenic effects *in vivo*

**Figure 6:**
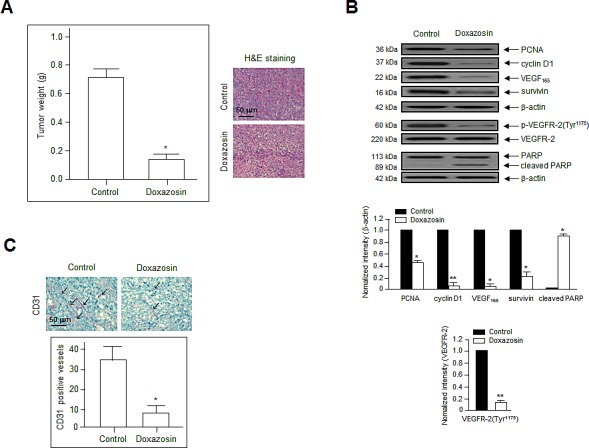
Doxazosin suppresses tumor growth by inhibiting angiogenesis *in vivo* (A) SKOV-3 ovarian cancer cells were injected subcutaneously into nude mice. (A) Mean tumor weight at the end of the experiments. *P<0.05 vs. the control group (left panel). Sections of doxazosin-treated and control tumors were stained with H&E (right panel). Bar = 50 μm. (B) Tumor tissues were evaluated by immunoblotting with primary antibodies against PCNA, cyclin D1, VEGF, survivin, p-VEGFR-2, and PARP. Data are representative of three independent experiments. Protein levels were calculated by densitometric analysis and normalized to levels of the loading control. *, *P*<0.05; **, *P*<0.01 compared with the controls. (C) CD31 staining was performed to visualize the blood vessels (arrows) in tumor tissues. Endothelial cells within frozen tumor sections were stained using anti-CD31 (PECAM-1) antibodies. Bar = 50 μm. *P<0.05 vs. the control.

We next determined whether doxazosin has direct effects on angiogenesis and tumor cell growth *in vivo*. SKOV-3 ovarian cancer cells were injected subcutaneously into nude mice (10/group), and the tumors were allowed to grow for 12 days until they reached a mean volume of 100 mm^3^. The mice were treated orally with control or doxazosin, and tumor growth and morphology was evaluated every 3 days for 24 days. Doxazosin-treated tumors weighed ~75% less than those from control mice (Fig. 6A, left panel).

Hematoxylin and eosin (H&E) staining revealed that tumors in the control group were high-grade carcinoma with an irregular cell distribution. In contrast, doxazosin-treated tumors exhibited large areas of late-apoptotic or necrotic cells (Fig. 6A, right panel). In addition, tumors treated with doxazosin had significantly reduced cell proliferation and enhanced apoptotic cell death, as determined by immunohistochemistry for PCNA and PARP, respectively (Fig. 6B). Moreover, anti-CD31 (PECAM-1) staining in the endothelial cells of doxazosin-treated mice revealed a ~2.5-fold reduced number of blood vessels compared to the control (Fig. 6C). These results suggest that doxazosin decreases tumor growth by suppressing angiogenesis *in vivo*.

## DISCUSSION

In this study, we identified a novel biological function of doxazosin as a powerful antiangiogenic modulator that functions via the Akt/mTOR signaling pathway. Quinazoline-derived α1-adrenoceptor antagonists exert anticancer activity in prostate cancer. In addition, the induction of apoptotic cell death and inhibition of angiogenesis by doxazosin have been reported widely [12-16]. Nevertheless, its precise molecular effects have not yet been reported in ovarian cancer. The phosphorylation of VEGFR-2 plays an important role in promoting VEGF-induced tumor angiogenesis. During cancer progression, angiogenesis is typically regulated by two key factors: VEGF, and HIF transcription factors. HIF proteins commonly enhance the expression of VEGF, whereas HIF-1α expression is enhanced during hypoxia [12]. Activated HIF-1α promotes the proliferation, migration, and invasion of endothelial cells, as well as tube formation in malignant cancer. The upregulation of VEGF is commonly seen in most aggressive solid tumors, including ovarian, colon, lung, and uterine tumors; it is closely associated with tumor progression and a poor prognosis [18-21]. However, the current study revealed that doxazosin could decrease both HIF-1α and VEGF expression during angiogenesis (Figs. 2D and 4). Treatment with doxazosin also suppressed VEGF-induced HUVEC proliferation significantly compared to the VEGF-treated control (Fig. 1A). Subsequently, we confirmed that doxazosin dramatically reduced the phosphorylation of PI3K and mTOR, as well as VEGFR-2 protein expression (Figs. 2A, 2D, and 5A). These results clearly suggest that doxazosin inhibits endothelial cell angiogenesis during tumorigenesis.

PI3K/Akt signaling plays a vital role in the biological functions of human malignant tumors. Recent studies have reported that suppressing PI3K might be beneficial to inhibit tumor angiogenesis [17, 22-24]. PI3K also regulates the signaling pathways that are involved in cell growth and/or apoptotic cell death [21]. Therefore, the anti-apoptotic events that are modulated by Akt begin with PI3K. Akt is activated by PI3K, which recruits Akt to the cell membrane and allows its phosphorylation by PDK1 [25]. The current study revealed that doxazosin decreased the phosphorylation of both PDK1 and Akt (Fig. 2). In addition, doxazosin downregulated the VEGF-induced phosphorylation of the mTOR signaling mediators p70S6K and 4E-BP1. In contrast, VEGF-induced ERK1/2 phosphorylation was unaffected by doxazosin (Fig. 3). Collectively, these results suggest that doxazosin could block VEGFR-2 transcriptional activity by suppressing VEGFR-2 phosphorylation via VEGF-dependent Akt/mTOR signaling.

In summary, our data demonstrate that doxazosin could suppress endothelial cell functions, including cell proliferation, migration, invasion, and capillary-like tubule formation, by suppressing VEGFR-2 phosphorylation and inhibiting Akt/mTOR signaling. It also suppresses the expression of HIF-1α and VEGF in ovarian carcinoma cells. These data supply additional evidence to support a role for doxazosin as a potent modulator of the biological and physiological mechanisms relevant to angiogenesis. The potential of doxazosin as an ovarian cancer treatment should be assessed in future studies.

## MATERIALS AND METHODS

### Cell lines, chemical, and antibodies

Human ovarian cancer cell lines (SKOV-3 and OVCAR-3) were purchased from the American Type Culture Collection (ATCC, Manassas, VA). They were grown in Dulbecco's modified Eagle's medium (DMEM; Life Technologies, Gaithersburg, MD) supplemented with 10% fetal bovine serum (FBS) and 100 U/ml penicillin/streptomycin at 37°C in a humidified 5% CO_2_ incubator. Primary HUVECs (Clonetics, San Diego, CA) were grown on 0.3% gelatin-coated dishes (Sigma-Aldrich, St. Louis, MO) in EGM-2 BulletKit medium (Clonetics). Rapamycin was purchased from Cell Signaling Technology (Beverly, MA). Doxazosin and all other chemicals were purchased from Sigma-Aldrich. The following primary antibodies were used: anti-phospho-VEGFR-2 (Y1175), anti-VEGFR-2, anti-phospho-PI3K, anti-PI3K, anti-phospho-Akt, anti-Akt, anti-HIF-1α, anti-phospho-ERK1/2, anti-ERK1/2 (all from Santa Cruz Biotechnology, Santa Cruz, CA), anti-phospho-PDK1, anti-PDK1, anti-phospho-4E-BP1, anti-4E-BP1, anti-phospho-mTOR, anti-mTOR, anti-phospho-p70S6K, anti-p70S6K, PCNA, cyclin D1, survivin (all from Cell Signaling Technology), anti-CD31 (Abcam, Cambridge, UK), anti-VEGF_165_ (Ab-1; Oncogene, Cambridge, MA), and anti-β-actin (Sigma-Aldrich).

### [^3^H]-Thymidine incorporation

[^3^H]-Thymidine incorporation was assessed as described previously [26, 27]. Briefly, HUVECs were plated into gelatin-coated plates at a density of 1.6 × 10^4^ cells/well in DMEM containing 10% FBS and 1% penicillin/streptomycin on day 0. After 18 h, the cells were rinsed twice with M199 and then incubated in M199 containing 1% FBS for 6 h. Cells were first incubated with doxazosin, and were then induced with VEGF (10 ng/ml, R&D Systems, Minneapolis, MN) for 24 h in M199 containing 1% FBS. [^3^*H*]-Thymidine (0.5 μCi/ml; Amersham, Arlington, IL) was added 4 h prior to analysis. High-molecular-mass compounds with [^3^H]-radioactivity were then precipitated using 10% trichloroacetic acid for 1 h at 4°C. Cells were solubilized in 0.2 N NaOH containing 0.1% sodium dodecyl sulfate (SDS), and [^3^H] thymidine incorporation was calculated using a liquid scintillation counter (Beckman Coulter, Franklin Lakes, NJ).

### Cell migration analysis

Twenty-four well Transwell chambers (8.0 μm pore size; Costar, New York, NY) were used to assay migration and invasion [26, 27]. For migration analyses, the lower surface of a filter was coated with 10 μg/ml of gelatin overnight. M199 containing 1% FBS and 10 ng/ml VEGF was added to the lower wells. Cells were harvested by trypsinization and washed. Next, 1.3 × 10^5^ cells were resuspended in 100 μl of fresh DMEM, added to the upper chamber, and incubated at 37°C for 24 h. Cells that had migrated to the lower chamber were fixed with methanol, and stained with 10 mg/ml H&E. Cells that remained on the surface of the upper filter were removed by wiping with a cotton swab. Cell migration was then quantified by counting the number of stained cells in five random areas of each membrane.

### *In vitro* tube formation assay

Growth factor-reduced Matrigel was added to a 24-well plate and polymerized for 30 min at 37°C [27]. Untreated, mock-treated, or doxazosin-treated HUVECs (3.3 × 10^5^ cells/well) were then added to the surface of the Matrigel. The seeded cells were incubated for 48 h with or without 10 ng/ml of VEGF in M199 containing 1% FBS. Images were then captured at 40× magnification after washing. The length of the formed tubes was measured using an inverted microscope equipped with a digital CCD camera and ImageLab software (MCM Design, Hillerød, Denmark). The control sample (VEGF-induced control) was defined as 100% tube formation, and the percent increase or decrease in tube formation relative to the control was measured for each sample.

### Analysis of PI3K activity

*In vitro* kinase assays were performed as described previously [28-30]. Briefly, cells were seeded at a density of 1.4 × 10^6^ cells/well. After an overnight incubation, the cells were treated with various concentrations of doxazosin for 6 h and then lysed in 1% NP-40 lysis buffer containing 20 mM Tris-HCl (pH 7.5), 100 mM NaCl, 1 mM EDTA, 1 mM MgCl_2_, 1% NP-40, 1 mM phenylmethylsulfonyl fluoride (PMSF), and 0.1 mM sodium orthovanadate. After the removal of insoluble materials by centrifugation, the supernatants were incubated at 4°C for 1 h with anti-p85 antibodies, followed by protein A-agarose beads for an additional 1 h at 4°C. The resulting immunoprecipitates were incubated in a kinase reaction buffer mixture containing 200 μg/ml phosphatidylinositol 3-phosphate and 2 μCi of [-32P] ATP for 15 min at 37°C. The reaction products were developed using autoradiography, and the radioactive lipids were quantified using liquid scintillation counting.

### Western blotting

Cells were rinsed with phosphate-buffered saline (PBS) and lysed in radioimmunoprecipitation assay (RIPA) buffer supplied with protease inhibitors. The concentration of protein samples was then evaluated using a Bradford protein assay kit (Bio-Rad, Hercules, CA). Equal amounts of protein were loaded onto 8-12% SDS-polyacrylamide gel electrophoresis (PAGE) gels and then transferred to polyvinylidene difluoride (PVDF) membranes (Bio-Rad). After blocking, the membranes were incubated at room temperature with primary antibodies for 1 h. They were then rinsed three times in wash buffer, followed by incubation with the appropriate horseradish peroxidase (HRP)-conjugated secondary antibodies. The protein bands were developed using an enhanced chemiluminescence (ECL) detection system.

### Tissue analysis by immunohistochemistry and Western blotting

Tumor samples were collected from mouse xenografts and fixed in 10% neutral-buffered formalin (Sigma-Aldrich). Slides were then stained using H&E (Sigma-Aldrich) according to the manufacturer's instructions. For immunoblotting, protein lysates were prepared by homogenizing the frozen tumor tissues. Protein quantitation and immunoblotting were then performed as described above with 30-μg protein/sample. For immunohistochemistry, paraffin-embedded ovarian tumor tissues were serially sectioned into 5-μm slices. The prepared slides were then deparaffinized, rehydrated in xylene and graded alcohols, and then rinsed in PBS. The slides were also incubated in 5% hydrogen peroxide in methanol for 20 min to block endogenous peroxidase activity. Sections were incubated with a saturating concentration of anti-mouse CD31 (platelet-derived endothelial cell adhesion molecule; PECAM-1) (Abcam) antibody overnight at 4°C, followed by a streptavidin-peroxidase complex at room temperature for 1 h. Microvessel density (MVD) was quantified in five randomly selected individual tumor fields (at 40× magnification) per sample, and the number of microvessels was counted under a high-powered microscope (400× magnification). All immunochemical analyses were performed using an Axiophot 2 apparatus (Carl Zeiss MicroImaging Inc., Thornwood, NY).

### Statistical analysis

Results were analyzed statistically using Student's *t*-test for comparisons between two groups. Data are presented as the means ± SDs, or SEM for triplicate experiments. Statistical significance was defined as *P*<0.05. Values with 95% confidence (*P*<0.05) are depicted with an asterisk (*) on each graph.

## References

[1] Folkman J, Shing Y (1992). Angiogenesis. J. Biol. Chem.

[2] Risau W (1997). Mechanisms of angiogenesis. Nature.

[3] Ferrara N (2002). VEGF and the quest for tumour angiogenesis factors. Nat. Rev. Cancer.

[4] Yoshiji H, Gomez DE, Shibuya M, Thorgeirsson UP (1996). Expression of vascular endothelial growth factor, its receptor, and other angiogenic factors in human breast cancer. Cancer Res.

[5] Gavin TP, Robinson CB, Yeager RC, England JA, Nifong LW, Hickner RC (2004). Angiogenic growth factor response to acute systemic exercise in human skeletal muscle. J. Appl. Physiol.

[6] Kraus RM, Stallings HW, Yeager RC, Gavin TP (2004). Circulating plasma VEGF response to exercise in sedentary and endurance-trained men. J. Appl. Physiol.

[7] Sawano A, Takahashi T, Yamaguchi S, Aonuma T, Shibuya M (1996). Flt-1 but not KDR/Flk-1 tyrosine kinase is a receptor for placenta growth factor (PlGF), which is related to vascular endothelial growth factor (VEGF). Cell Growth Differ.

[8] Ferrara N (2004). Vascular endothelial growth factor: basic science and clinical progress. Endocr. Rev.

[9] Takahashi S (2011). Vascular endothelial growth factor (VEGF), VEGF receptors and their inhibitors for antiangiogenic tumor therapy. Biol. Pharm. Bull.

[10] Shibuya M (2013). Vascular endothelial growth factor and its receptor system: physiological functions in angiogenesis and pathological roles in various diseases. J. Biochem.

[11] Breier G (2000). Endothelial receptor tyrosine kinases involved in blood vessel development and tumor angiogenesis. Adv. Exp. Med. Biol.

[12] Ferrara N (2000). Role of vascular endothelial growth factor in regulation of physiological angiogenesis. Am J Physiol Cell Physiol.

[13] Meyer RD, Rahimi N (2003). Comparative structure-function analysis of VEGFR-1 and VEGFR-2: What have we learned from chimeric systems? Ann. N Y Acad. Sci.

[14] Meyer RD, Singh A, Majnoun F, Latz C, Lashkari K, Rahimi N (2004). Substitution of C-terminus of VEGFR-2 with VEGFR-1 promotes VEGFR-1 activation and endothelial cell proliferation. Oncogene.

[15] Kirby RS, Pool JL (1997). α-adrenoceptor blockade in the treatment of benign prostatic hyperplasia: past, present and future. Br. J. Urol.

[16] Benning CM, Kyprianou N (2002). Quinazoline-derived α1-adrenoceptor antagonists induce prostate cancer cell apoptosis via a α1-adrenoceptor-independent action. Cancer Res.

[17] Tahmatzopoulos A, Rowland RG, Kyprianou N (2004). The role of α-blockers in the management of prostate cancer. Expert Opin. Pharmacotherapy.

[18] Partin JV, Anglin IE, Kyprianou N (2003). Quinazoline-based α1-adrenoceptor antagonists induce prostate cancer cell apoptosis via TGF-β1 signaling IκBα induction. Br. J. Cancer.

[19] Keledjian K, Kyprianou N (2003). Anoikis induction by quinazoline based α1-adrenoceptor antagonists in prostate cancer cells: antagonistic effect of bcl-2. J. Urol.

[20] Garrison JB, Kyprianou N (2006). Doxazosin induces apoptosis of benign and malignant prostate cells via a death receptor-mediated pathway. Cancer Res.

[21] Jiang BH, Liu LZ (2008). AKT signaling in regulating angiogenesis. Curr. Cancer Drug Targets.

[22] Olson TA, Mohanraj D, Carson LF, Ramakrishnan S (1994). Vascular permeability factor gene expression in normal and neoplastic human ovaries. Cancer Res.

[23] Paley PJ, Staskus KA, Gebhard K, Mohanraj D, Twiggs LB, Carson LF, Ramakrishnan S (1997). Vascular endothelial growth factor expression in early stage ovarian carcinoma. Cancer.

[24] Ishigami SI, Arii S, Furutani M, Niwano M, Harada T, Mizumoto M, Mori A, Onodera H, Imamura M (1998). Predictive value of vascular endothelial growth factor (VEGF) in metastasis and prognosis of human colorectal cancer. Br. J. Cancer.

[25] Ohta Y, Tomita Y, Oda M, Watanabe S, Murakami S, Watanabe Y (1999). Tumor angiogenesis and recurrence in stage I non-small cell lung cancer. Ann. Thorac. Surg.

[26] Meng Q, Xia C, Fang J, Rojanasakul Y, Jiang BH (2006). Role of PI3K and AKT specific isoforms in ovarian cancer cell migration, invasion and proliferation through the p70S6K1 pathway. Cell Signal.

[27] Xia C, Meng Q, Cao Z, Shi X, Jiang BH (2006). Regulation of angiogenesis and tumor growth by p110 alpha and AKT1 via VEGF expression. J. Cell Physiol.

[28] Arbiser JL, Kau T, Konar M, Narra K, Ramchandran R, Summers SA, Vlahos CJ, Ye K, Perry BN, Matter W, Fischl A, Cook J, Silver PA, Bain J, Cohen P, Whitmire D, Furness S, Govindarajan B, Bowen JP (2007). Solenopsin, the alkaloidal component of the fire ant (Solenopsis invicta), is a naturally occurring inhibitor of phosphatidylinositol-3-kinase signaling and angiogenesis. Blood.

[29] Duronio V, Scheid MP, Ettinger S (1998). Downstream signalling events regulated by phosphatidylinositol 3-kinase activity. Cell Signal.

[30] Lee OH, Kim YM, Lee YM, Moon EJ, Lee DJ, Kim JH, Kim KW, Kwon YG (1999). Sphingosine 1-phosphate induces angiogenesis: its angiogenic action and signaling mechanism in human umbilical vein endothelial cells. Biochem. Biophys. Res. Commun.

[31] Rho SB, Song YJ, Lim MC, Lee SH, Kim BR, Park SY (2012). Programmed cell death 6 (PDCD6) inhibits angiogenesis through PI3K/mTOR/p70S6K pathway by interacting of VEGFR-2. Cell. Signal.

[32] Fruman DA, Mauvais-Jarvis F, Pollard DA, Yballe CM, Brazil D, Bronson RT, Kahn CR, Cantley LC (2000). Hypoglycaemia, liver necrosis and perinatal death in mice lacking all isoforms of phosphoinositide 3-kinase p85 alpha. Nat. Genet.

[33] Franch HA, Wang X, Sooparb S, Brown NS, Du J (2002). Phosphatidylinositol 3-kinase activity is required for epidermal growth factor to suppress proteolysis. J. Am. Soc. Nephrol.

[34] Rho SB, Kim BR, Kang S (2011). A gene signature-based approach identifies thioridazine as an inhibitor of phosphatidylinositol-3'-kinase (PI3K)/AKT pathway in ovarian cancer cells. Gynecol. Oncol.

